# Effect of Cobalt and Chromium Content on Microstructure and Properties of WC-Co-Cr Coatings Prepared by High-Velocity Oxy-Fuel Spraying

**DOI:** 10.3390/ma16217003

**Published:** 2023-11-01

**Authors:** Qun Wang, Yuan Zhong, Haifeng Li, Shaoyi Wang, Jianwu Liu, Yiwei Wang, Chidambaram Seshadri Ramachandran

**Affiliations:** 1 Department of Metal Materials, College of Materials Science and Engineering, Hunan University, Changsha 410082, China; yuanzhong@hnu.edu.cn; 2 Transmission R&D Center, AECC Hunan Aviation Powerplant Research Institute, Zhuzhou 412002, China; lihaifeng3737@163.com (H.L.); 18142620191@163.com (J.L.); wyw9199@163.com (Y.W.); 3 Chongyi Zhangyuan Tungsten Industry Co., Ltd., Chongyi 341300, China; zywsy@achteck.com.cn; 4 Department of Materials Science and Engineering, The State University of New York (SUNY), New York, NY 11794 2275, USA; csrcn1@gmail.com

**Keywords:** high-velocity oxygen fuel spraying, WC-Co-Cr, abrasive wear, corrosion

## Abstract

To explore the Co/Cr ratio impact on the high-velocity oxygen fuel (HVOF)-sprayed WC-Co-Cr coatings microstructure and performances, three kinds of WC-Co-Cr coatings, namely WC-4Co-10Cr, WC-7Co-7Cr, and WC-10Co-4Cr, were prepared by using a high-velocity oxygen fuel (HVOF) spraying process. The three coatings’ phase composition, microstructure, basic mechanical properties, abrasive wear, and corrosion resistance were investigated. The results show that all three WC-Co-Cr coatings comprise the main phase WC, minor W_2_C, and amorphous W-Co-Cr phase, besides the WC-4Co-10Cr coating containing a small amount of Cr_x_C_y_ phase. In addition, WC-7Co-7Cr coating exhibited the highest hardness and abrasive wear resistance, followed by WC-10Co-4Cr and WC-4Co-10Cr coatings. The corrosion resistance as a hierarchy was found to be WC-10Co-4Cr > WC-7Co-7Cr > WC-4Co-10Cr.

## 1. Introduction

Tungsten-carbide (WC)-based powders are widely used in high-velocity oxygen fuel (HVOF) spraying to produce compact coatings with high hardness and excellent wear and corrosion resistance that are commonly used in petroleum, mining, metallurgy, paper, and aviation industries [1,2,3]. Carbide-based materials widely used for HVOF spraying include WC-Co, WC-Co-Cr, WC-Ni, WC-Cr_3_C_2_-Ni, and Cr_3_C_2_-Ni, wherein the WC-Ni coating is often used to improve the wear resistance of the surface of parts with impact. WC-Cr_3_C_2_-Ni, Cr_3_C_2_-NiCr coatings are also used to resist wear, and their service ambient temperatures are not higher than 650 °C and 875 °C, respectively. The most frequently used cermet coatings deposited using HVOF-spraying are WC-Co and WC-Co-Cr below 500 °C [4]. Compared with WC-Co coating, the WC-Co-Cr deposit has higher corrosion resistance because it has the Cr, which can generate a passivation layer on its surface in a corrosive environment [4,5,6]. This passivation film structure exists on the surface of the WC-based coatings containing chromium in the form of oxides, and its structure is relatively dense, which can isolate the corrosive medium from the metal-binding phase, effectively inhibiting the dissolution of the binding material into the solution [4]. It was reported that the WC coating containing chromium prepared by the HVOF process is superior to the electroplating hard chromium coating in both NaCl [7] and HCl solutions [8] and can replace the electroplating hard chromium process, which will cause severe pollution to the environment. Generally, the chemical composition of the conventional WC-Co-Cr is WC-10Co-4Cr; that is, the mass% of WC, Co, and Cr are 86%, 10%, and 4%, respectively. Since adding Cr can improve the corrosion resistance of WC-based coatings [9], the threshold level of Cr content in the layers needed for improving the coating performance has not been investigated yet. Therefore, in this work, three kinds of WC-Co-Cr powder, namely, the conventional WC-10Co-4Cr and two new types of WC-Co-Cr powders, namely, WC-7Co-7Cr and WC-4Co-10Cr, were sprayed on low-carbon steel substrate by using HVOF spraying, and their phase composition, microstructure, abrasive wear, and corrosion performances were compared comprehensively.

## 2. Experiment

### 2.1. Materials

The three kinds of WC-Co-Cr powders were spray-dried, agglomerated, sintered, crushed, and sieved to obtain the sprayable spherical particles with identical size distributions (Supplier: Chongyi Zhangyuan Tungsten Co., Ltd., Ganzhou, China). Although finer powder can achieve higher coating hardness, its flowability is poor and tends to melt over, resulting in nozzle clogging. In comparison, coarser powder will result in a lower hardness of the coating. The apparent density of the WC-based powder has a significant effect on the coating performance; namely, too-low powder apparent density will cause the powder to overmelt in the barrel, while too-high apparent density will cause the powder deposition rate to be low. Therefore, the size distribution of 15–45 µm and medium apparent density of ~5 g/cm^3^ of three kinds of WC-Co-Cr powders with good flow ability were adopted in this work. Their chemical compositions and basic portieres are shown in Table 1.

From Table 1, all the WC-Co-Cr powders exhibited similar basic properties except for the different Cr/Co binder ratios. 

### 2.2. Coating Preparation

All the WC-Co-Cr powders were sprayed on a rinsed and sandblasted low-carbon steel substrate (16Mn steel, its nominal mass percentage content, C: 0.13–0.19, Si: 0.20–0.60, Mn: 1.20–1.60, Cr ≤ 0.30, P ≤ 0.030, S ≤ 0.030, Ni ≤ 0.30, Cu ≤ 0.25, Anshan Iron and Steel Group Co., LTD., Anshan, Liaoning, China) using Praxair-JP-8000 HVOF equipment (Praxair Surface Technologies, INC, Indianapolis, IN, USA) with balanced and identical spray parameters (Table 2) [10]. 

The barrel was perpendicular to the surface of the substrate; the spray gun was traversed along the horizontal direction according to the Z-shaped trajectory. Because the thickness of the sprayed 16Mnsteel substrate was only 8 mm, and the expansion coefficient between the coating and the substrate was also different, long-term spraying would make the temperature of the substrate too high, which would bring great residual stress to the deposited layer, and too high a thermal stress can cause the sample to bend or even the coating to crack; therefore, the substrate temperature was kept below 160 °C during the spraying process by intermittent pause time and compressed air cooling. 

### 2.3. Characterization

X-ray diffraction (XRD) analysis of the powders and coatings was performed with a Rigaku D/max-2550 diffraction meter (Rigaku Corporation, Tokyo, Japan) using Cu-Kα radiation (λ = 1.5418 Å), which operated at the voltage and current of 40 kV and 15 mA, respectively. The scanning speed was 5°/min, the step size was 0.02°, and the scanning range was 10°–90°. The scanning electron microscope (SEM; MIRA3 LMH, Tescan Orsay Holding, a.s., Brno, Czech Republic) with EDS (Ultim Max 20, Oxford Instruments, Abingdon, UK) examined the cross-sectional microstructure morphologies of the three WC-Co-Cr powders and coatings. Both porosity and hardness measurements were performed on the polished cross-sections of the as-sprayed WC-10Co-4Cr coatings. The coating porosity was measured by the grayscale method. It is worth noting that when preparing cross-section samples for measuring the porosity of the HVOF-sprayed WC-based coatings, it was recommended to use diamond sandpaper (Beijing Guoruisheng Technology Co., LTD, Beijing, China) and diamond spray (Shanghai Yunbo Testing Technology Co., LTD, Shanghai, China) for grinding and polishing. The polishing time should not be too long because a long polishing would make the WC particles in the coating fall off, so the measured coating porosity was higher than the actual porosity of the layer. The coating hardness was measured under 0.3 kg and 5 kg, respectively. Cracks parallel to the interface between coating and substrate on the cross-sections of the as-sprayed layers under the load of 5 kg. After measuring the indentation’s crack length and the diagonal length, the fracture toughness was calculated according to the Evans and Wilshaw equation [11]. It is worth noting that when measuring the fracture toughness, the indenter needs to be pressed in the middle of the coating thickness direction, and one of the diagonals of the indentation should be parallel to the interface between the coating and the substrate. In addition, if the thickness of the coating being measured is different, one must try to keep the center of the indentation the same distance from the surface of the layer. Ten points are calculated when measuring the above coating properties, such as porosity, hardness, and fracture toughness, and an average of ten readings were reported.

### 2.4. Abrasive Wear

The abrasive wear resistance of all the coatings was evaluated according to the ASTM G105 (Standard Test Method for Conducting Wet Sand/Rubber Wheel Abrasion Tests). The load was 100 N, and each sample’s total revolutions was 3000 r. The slurry used was composed of 1.5 kg SiO_2_ sand (Changsha Huanyu Quartz Sand Co., LTD, Changsha, China) with a particle size of 40–70 mesh and 1 kg fresh water; the mass difference before and after the wear test was divided by the theoretical density of the coatings as well as the total wear distance and loading, which were used to calculate the wear rate of each coating. In addition, the worn surface morphologies of the three coatings were examined by scanning electron microscopy (SEM: MIRA3 LMH, TESCAN) to explore their abrasive wear mechanism.

### 2.5. Electrochemical Corrosion

Electrochemical corrosion was conducted with 3.5% NaCl solution at 25 °C in an electrochemical workstation (CHI 604D, Chen-Hua Instruments Co., Ltd., Shanghai, China) that had a 3-electrode configuration (ASTM G69–20). The coating sample was used as the working electrode, and a platinum plate and a saturated calomel electrode were used as the counter and reference electrodes in the corrosion testing. In addition, the corrosion surface morphologies of the three coatings after electrochemical corrosion were examined by the MIRA3 LMH, TESCAN scanning electron microscopy with an Energy Dispersive Spectrometer (Ultim Max 20), to explore their corrosion mechanism.

## 3. Results and Discussion 

### 3.1. Cross-Sectional Morphologies of the Three WC-Co-Cr Powders

**Figure 1:**
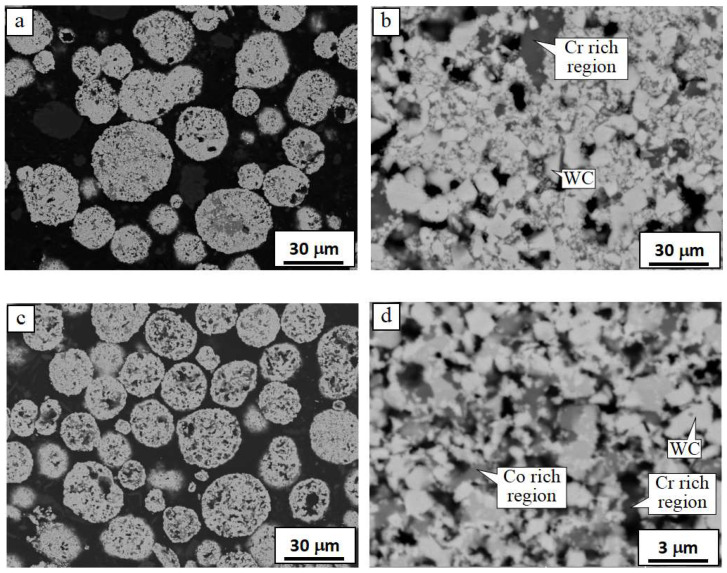

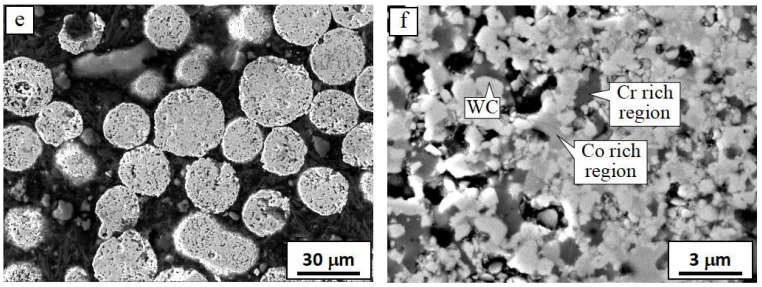
Cross-sectional morphologies of the (**a**,**b**) WC-4Co-10Cr, (**c**,**d**) WC-7Co-7Cr, and (**e**,**f**) WC-10Co-4Cr powders.

The cross-sectional morphologies of the three WC-Co-Cr powders are shown in Figure 1.

As observed in Figure 1, the spray powder particles were of spherical or subspherical shape (Figure 1a,c,e), which had good flowability and were well fed through the powder feeding system. Metallic Co-Cr was bonded with polygonal WC particles to form a spherical powder particle. The dark and gray regions are supposed to be rich in Cr and Co, respectively, according to the backscattered electrons (BSE) images shown in Figure 1b,d,f. These cermet powders were prepared by the agglomerated sintering method and the manufacturing process was as follows. Firstly, the original WC, Co, and Cr particles with the size of microns, as well as a certain amount of deionized water or alcohol and organic binders, such as Polyvinyl alcohol (PVA, Shanghai Maclin Biochemical Technology Co., LTD, Shanghai, China) or Polyethylene glycol (PEG, Shanghai Merrier Biochemical Technology Co., LTD, Shanghai, China), were mixed and ball milled to obtain a mixture of slurry, which was then sprayed to form spherical particles. Then, the agglomerated particles were sintered, crushed, and sieved to produce WC-Co-Cr cermet powder with suitable apparent density and particle size distribution, which was ideal for HVOF spraying. 

### 3.2. Phase Composition

**Figure 2:**
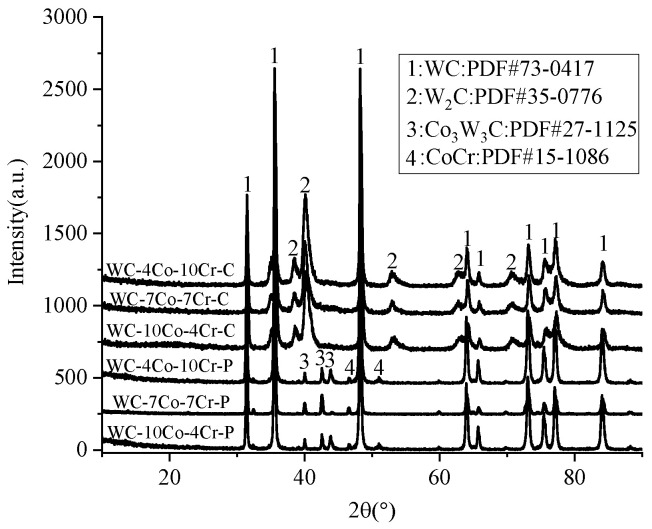
X-ray diffraction (XRD) patterns of WC-Co-Cr powders and resultant coatings (the -P and -C at the end of each curve indicate powder and coating, respectively).

XRD results of WC-Co-Cr powder and resultant coatings produced by the HVOF spray process are shown in Figure 2.

Observed from Figure 2, all the WC-Co-Cr coatings showed almost the same XRD patterns with the main WC, minor W_2_C, and amorphous Co-W-C binder. Compared with their starting powder, the Co and Co_3_W_3_C phases have disappeared and formed amorphous Co-W-C (2θ: 36°–46°) in all the coatings due to the rapid cooling rate of molten and semi-molten sprayed particles when they impacted on the substrate during the spraying process [4,12]. All the phase content of both WC-Co-Cr powers and coatings are shown in Table 3.

Table 3 shows that decarburization had occurred for all three WC-Co-Cr coatings during the HVOF spraying as a reason for generating various content of the W_2_C phase. There are two ways of WC decarburization during the HVOF spraying of WC-based powders. Namely, WC particles react directly with oxygen, leading to direct decarburization. WC and WC particles reacted with oxygen from liquid cobalt, resulting in indirect decarburization [12]. Therefore, it can be concluded that direct decarburization dominated the HVOF process in this work because the W_2_C content increased with the Cr content of the original feedstocks (Table 2). For example, WC-4Co-10Cr exhibited the highest decarburization, which can be attributed to the lack of cobalt, leading to a large number of WC particles being directly exposed to an oxygen-containing atmosphere in the HVOF flame flow, resulting in a large amount of direct decarburization, and vice versa.

### 3.3. Microstructure

**Figure 3:**
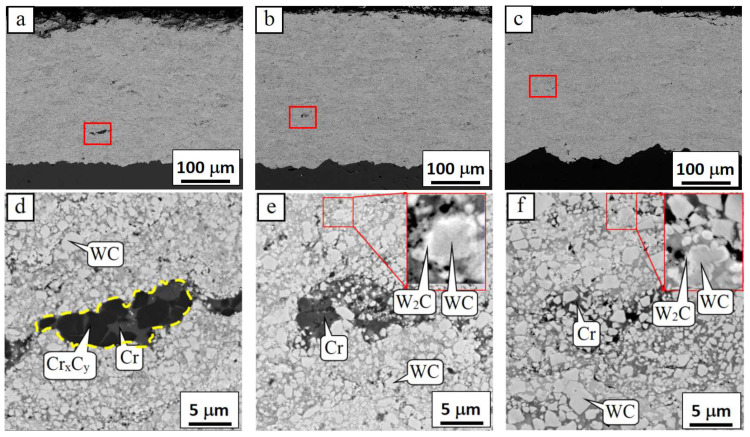
SEM images of the cross-sectional of the (**a**,**d**) WC-4Co-10Cr, (**b**,**e**) WC-7Co-7Cr, and (**c**,**f**) WC-10Co-4Cr coatings ((**d**–**f**) are the enlarged images of the red box areas shown in (**a**–**c**), respectively).

Low- and high-magnification BSE images of the cross-sectional of the three WC-Co-Cr coatings are shown in Figure 3.

**Figure 4:**
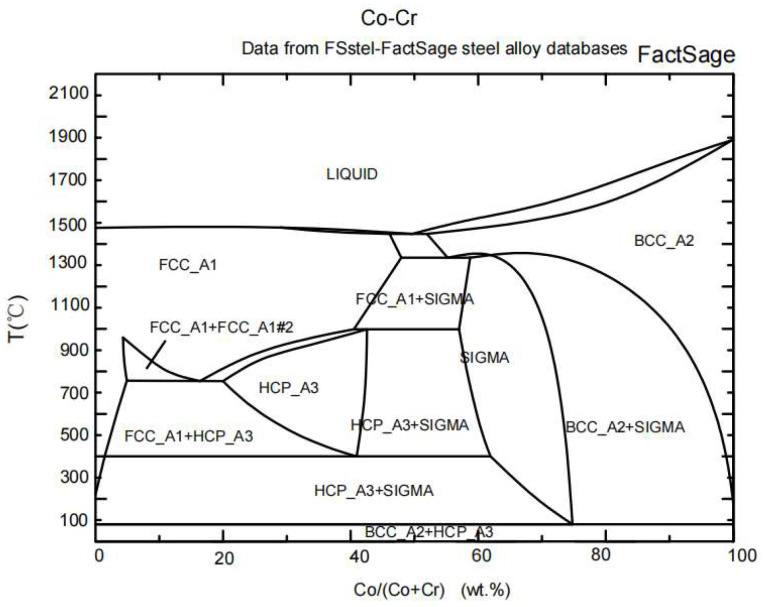
Co-Cr binary phase diagram (BCC: body-centred cubic, FCC: face-centred cubic, HCP: hexagonal close packing, Co: Cobalt, Cr: Chrome).

From Figure 3, it is seen that all the coatings are tightly bound to the substrate with a compact microstructure. The WC-4Co-10Cr coating with the highest Cr content showed the most prominent dark area, as well as WC-7Co-7Cr and WC-10Co-4Cr. The dark area in the BSE images of all the WC-Co-Cr coatings should be Cr and Cr_x_C_y_. To add Cr particles, they are more likely to be ground into a long strip shape than fine powder in the wet-grinding mixing stage in the powder preparation process. The binder Co, however, exhibited uniform distribution among the main phase WC. Most WC particles retained their polygonal shape, except for a few surrounded by a circle of white material. The reasons for generating this kind of microstructure of WC-Co-Cr coating may be explained by using the Co-Cr binary phase diagram shown in Figure 4.

From Figure 4, the Co and CoCr alloys of molten powder particles are liquid, while the Cr and WC particles are always solid. Due to the very short residence time of powder particles in the plume before they impact the substrate, the temperature inside some particles, massive particles, is lower than 1741–1890 °C [13], leading to a lower degree of powder melting. Therefore, nearly all the molten Co in WC-Co-Cr is a rapidly solidified structure and is in an amorphous state. A broad maximum between 38° and 48° of 2θ can be found in Figure 2 for all the as-sprayed WC-10Co-4Cr coatings, which is the characteristic peak of amorphous materials, confirming the generation of an amorphous phase. Very limited unmolten Co is still in the hexagonal close-packed (HCP) structure, resulting in its homogeneous distribution in the as-sprayed layers. As for Cr, the existence state is relatively complex, and there are three primary states of Cr existence: (1) maintaining an elemental state with BCC structure, (2) forming an amorphous CoCr alloy, and (3) forming Cr_y_C_x_ with C diffused from WC due to the excess Cr reacted with C diffused from WC into the metal-binding phase to form Cr_x_C_y_ in high-temperature flame. Since the residence time of powder particles in flame is limited, most Cr_x_C_y_ generated were Cr_23_C_6_ and Cr_7_C_3_ with a small amount. The area marked by the yellow dashed line in Figure 3d exhibited two kinds of phase; the black and grey particles are supposed to be Cr_x_C_y_ and Cr phases, respectively. In addition, the inserted high magnification image in Figure 3e,f shows that a circle of white phase surrounds WC particles. According to numerous pieces of the literature, the generation of the W_2_C around WC is attributed to the decarbonization of WC during the HVOF process [4,13,14]. Some researchers proposed that the decarbonization of WC caused by the diffusion of C in WC to the surrounding liquid-phase Co is the most crucial decarbonization form in the HVOF process, called indirect decarbonization [15,16,17]. Therefore, under the same spraying parameters, the WC-10Co-4Cr and WC-7Co-7Cr powders with higher Co content are more likely prone to indirect decarbonization and generating the W_2_C compared with WC-4Co-10Cr, which can also be illustrated by XRD results (Figure 2). 

### 3.4. Basic Mechanical Properties

The basic mechanical properties of the three WC-Co-Cr coatings are shown in Table 4.

Table 4 shows that WC-4Co-10Cr showed the lowest hardness and fracture toughness but the highest porosity among all three coatings, which can be attributed to its limited content of Co binder. Because the Co has better plasticity and wettability with WC [18], as well as a finer size and lower melting-point temperature compared with Cr, the Co-Cr binder in the WC-7Co-7Cr and WC-10Co-4Cr powders are more likely to melt and quickly fill the gap between WC, resulting in a compact coating microstructure with high hardness compared with that of WC-4Co-10Cr. From Table 4, it can also be found that the fracture toughness of the WC-Co-Cr coatings decreased with the increase in the Cr content. The reason is as follows: because Cr has higher hardness and poorer wettability with WC in comparison to Co [18], it will undoubtedly increase the brittleness of the metal-binding phase and decrease the bonding force between the metal-binding phase and WC; therefore, the cracks in the coating are more accessible to propagate under the action of the indenter during the testing of fracture toughness. In addition, the fracture toughness obtained in this work is comparable to those of the WC-10Co-4Cr coatings reported in the other literature [4,7], but it is lower than that of the WC-Co coating for the same reasons as above [10].

### 3.5. Corrosion Resistance

**Figure 5:**
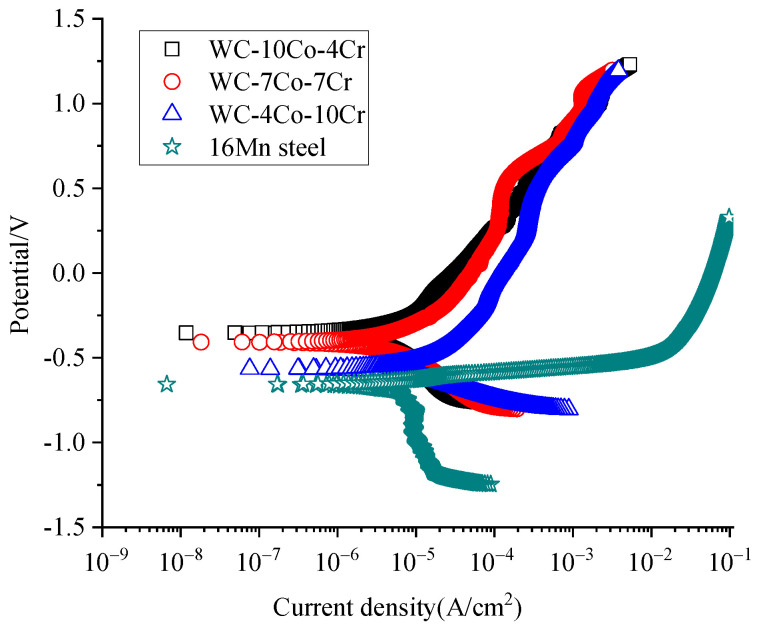
Polarization curves of the three WC-Co-Cr coatings and 16Mnsteel substrate in 3.5% NaCl solution.

The potentiodynamic polarization curves of the three kinds of HVOF-sprayed WC-Co-Cr coatings and 16Mnsteel substrate in the 3.5 wt.% NaCl solution are displayed in Figure 5.

The corrosion current density of the three WC-Co-Cr coatings calculated from Figure 4 is presented in Table 5. From Table 5, it can be found that all three HVOF-sprayed WC-Co-Co coatings exhibited similar polarization curve shapes in the tested NaCl solution, but their open circuit potential, corrosion potential, and corrosion current density were different; the WC-10Co-4Cr coating has the best corrosion resistance, followed by WC-7Co-7Cr, and WC-4Co-10Cr has the lowest open circuit potential and highest corrosion current density among all the three WC-Co-Cr layers, which shows the poorest corrosion resistance. Namely, the corrosion resistance of the HVOF-sprayed WC-Co-Cr coating decreased with the increase in the Cr in the Co-Cr binder, which was inconsistent with the corrosion resistance of the usual chrome-containing steels. The reasons for this abnormal corrosion result could be as follows. (1) The melting point of Cr is higher than that of Co, and Cr is intricate to ground into fine particles in ball milling. In addition, the affinity between Cr and WC is worse than that of Co. Therefore, when the Cr/Co ratio of the Co-Cr binder increases, the porosity of the resultant sprayed WC-Co-Cr coating will increase, which, on the one hand, will lead to an increase in the contact area between the coating and sodium chloride solution, and on the other hand, will also increase the infiltration of sodium chloride solution into the interface between the coating and the substrate through the penetrating pores, and ultimately lead to the decrease in the corrosion protection ability of the layer [4]. (2) Although Cr in the coating can form a passivation film in sodium chloride solution when Cr addition increases to 10 mass%, some of Cr will react with C to form Cr_x_C_y_, which reduces the effect of Cr forming a passivation film. (3) The WC phase in the WC-Co-Cr coating does not react with the sodium chloride solution, and the actual Cr content in the metal binder Co-Cr of the WC-10Co-4Cr coating is 28.57%. It is higher than the 12.5% Cr content, which can form a complete passivation film during the corrosion test. In addition, the WC-10Co-4Cr coating has the lowest porosity among all the WC-Co-Cr layers, which makes it the best to resist sodium-chloride-solution corrosion. Although the corrosion resistance of the three WC-Co-Cr coatings is different, their corrosion resistance is significantly better than that of the 16Mnsteel substrate because the 16Mnsteel substrate exhibited higher open-circuit and corrosion potential and lower corrosion current density than those of the as-sprayed WC-Co-Cr coatings.

**Figure 6:**
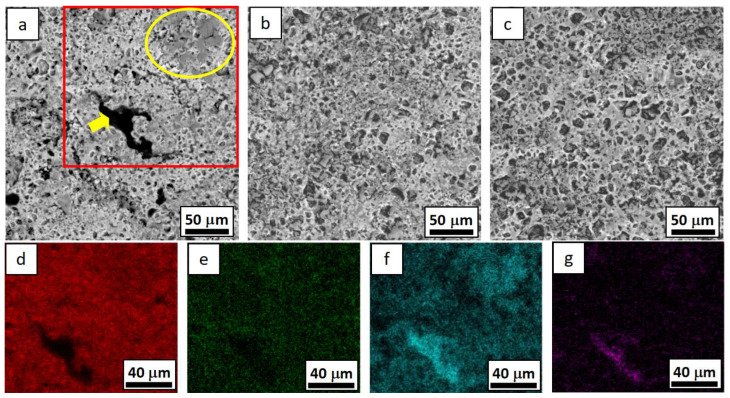
Corrosion surfaces of the coatings after electrochemical corrosion in static 3.5 wt.% NaCl solution: (**a**) WC-4Co-10Cr, (**b**) WC-7Co-7Cr, and (**c**) WC-10Co-4Cr coatings and EDS mapping of the area marked by red rectangle: (**d**) W, (**e**) Co, (**f**) Cr, (**g**) O.

The images of the three WC-Co-Cr coatings’ corrosion surfaces and EDS mapping after electrochemical corrosion in 3.5 wt.% NaCl solution is shown in Figure 6.

Figure 6 shows that many pits appeared on the corrosion surfaces of the three WC-Co-Cr coatings, which was supposed to be caused by the WC particles falling off during the corrosion process. The potential of WC was higher than that of the metal-binding phase. Therefore, many galvanic batteries were formed between the two phases, resulting in galvanic corrosion. This led to the preferential dissolution of the binding phase and detachment of WC particles during the electrochemical corrosion process. In addition, the surface of the coating of WC-4Co-10Cr has long strips of black material (marked by an arrow in Figure 6a), and gray coverings (marked by the yellow ellipse in Figure 6a) were supposed to be chromium-rich areas, according to the EDS mapping results shown in Figure 6d–g, due to its highest Cr content. Meanwhile, Cr-rich regions often correspond to high oxygen content (Figure 6d–g), indicating that chromium oxide is formed by the reaction of Cr with oxygen during corrosion testing. Although the high chromium content helps to generate more chromium oxide, the WC particles in some chromium-rich areas (marked by an arrow in Figure 6a) were not adequately coated by the metal binder phase due to the poor wettability of chromium and WC, resulting in electrolyte penetration into the internal pores of the coating, which finally led to more severe corrosion of WC-4Co-10Cr with high Cr content. Therefore, when designing the composition of the WC-Co-Cr coating, it is important to not only to consider the characteristics of Cr that can form a dense passivation film and improve the corrosion resistance of the layer but also to consider whether the wettability and affinity of Cr and WC are poor, and it is necessary to add enough Co with good wettability of WC to obtain a dense and high corrosion resistance of the cermet coating.

### 3.6. Abrasive Wear Resistance

**Figure 7:**
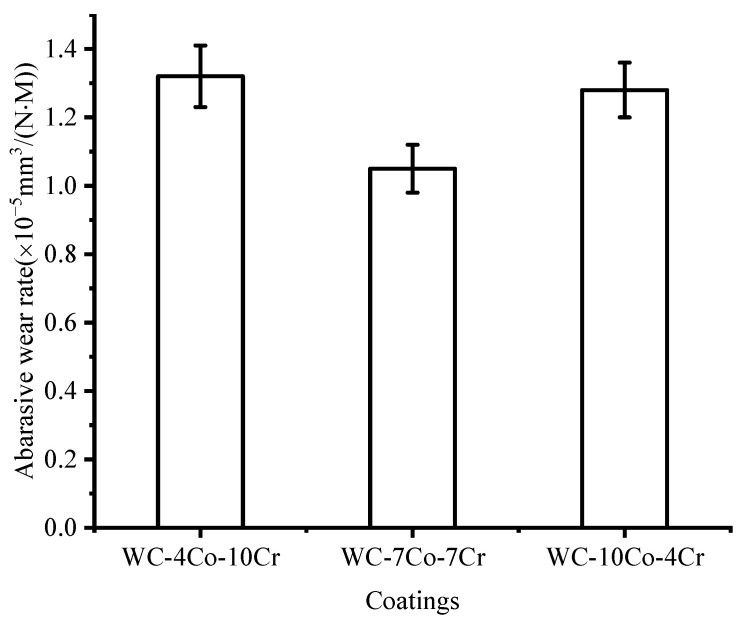
The abrasive wear rate of the three WC-Co-Cr coatings.

The abrasive wear rates of the three WC-Co-Cr coatings are shown in Figure 7. Figure 7 shows that WC-7Co-7Cr coating has the best abrasive wear resistance, followed by WC-10Co-4Cr and WC-4Co-10Cr, which have the lowest wear resistances.

**Figure 8:**
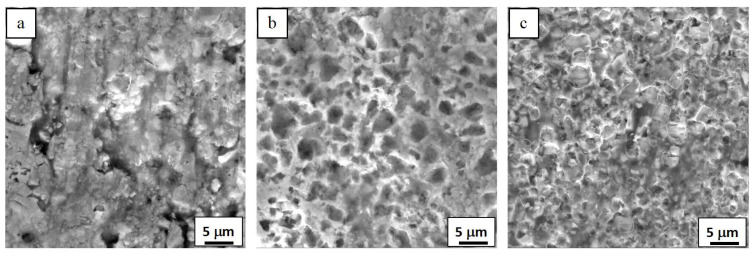
Abrasive wear surface morphologies of the HVOF-sprayed (**a**) WC-4Co-10Cr, (**b**) WC-7Co-7Cr, and (**c**) WC-10Co-4Cr.

The typical abrasive, worn surface morphologies of the HVOF-sprayed WC-Co-Cr coatings are shown in Figure 8.

The abrasive wear mechanism of WC-based coating accepted by most researchers is that the soft metallic binder phase is removed first. The WC particles are impacted by abrasives into fragments and removed from the surface of the coating at last [4,5,10,11,19]. According to the worn-surface micromorphologies observed in Figure 8, the above mechanism can still explain the wear failure process of the three WC-Co-Cr coatings tested in this work. From Figure 8a, it can be found that the WC-4Co-10Cr coating showed a coarser worn surface and more pronounced grooves than those of the other coatings. Because WC-4Co-10Cr has the lowest hardness and highest porosity, SiO_2_ particles driven by rubber wheels can easily penetrate those loose areas of the coating with low hardness, resulting in significant grooves and a high wear rate during the abrasive wear process. The WC-7Co-7Cr and WC-10Co-4Cr coatings have a dense microstructure and high hardness, which can effectively hinder the penetration of the SiO_2_ abrasive into the coatings [5,20]. Although there are no apparent grooves appearing on the worn surface of the WC-7Co-7Cr and WC-10Co-4Cr coatings, fatigue damage to the coating is inevitable; namely, the removal of the metallic binder phase, the cyclic fatigue, carbide grain fracture, the undermining of the particles and subsequent particle pullout were found to be the dominant wear-related failure processes for these two coatings according to their worn surfaces, shown in Figure 8b,c. In addition, the residual binding Co-Cr phase on the worn surface of the WC-7Co-7Cr coating is more than that of on the worn surface of the WC-10Co-4Cr coating (Figure 8b,c), which can be attributed to the high hardness Cr content (50 mass%) in the Co-Cr binder phase of the former coating is higher than that of the latter one (28.6 mass%). Therefore, the binder phase of WC-7Co-7Cr coating is removed by SiO_2_ abrasive particles more slowly than that of WC-10Co-4Cr coating during the process of the abrasive wear test result in that the wear rate of WC-7Co-7Cr coating is about 18% lower than that of the latter.

## 4. Conclusions

Three kinds of WC-Co-Cr powders and resultant coatings were prepared with changing Cr/Co ratio with constant WC (86 mass%) content. The microstructure, abrasive wear, and corrosion properties of the consequent HVOF-sprayed coatings were studied in detail, and the conclusions are as follows:

Although increasing the Cr/Co ratio of the Co-Cr binder will reduce the corrosion resistance of WC-Co-Cr coating, the corrosion resistance of WC-7Co-7Cr coating was only slightly less than that of the WC-10Co-4Cr coating; in addition, its abrasive wear resistance is 21.9% higher than that of conventional WC-10Co-4Cr coating, as the Cr/Co ratio is 1:1. When the Cr/Co ratio increased to 2.5, additional Cr_x_C_y_ phases were generated in the as-sprayed WC-4Co-10Cr coating with highest porosity and lowest fracture toughness, abrasive wear, and corrosion resistance. Therefore, WC-7Co-7Cr is supposed to replace conventional WC-10Co-4Cr coating when high wear resistance is required.

## Figures and Tables

**Table 1:** Nominal chemical composition and basic portieres of the three WC-Co-Cr powders.

Powder	Chemical Composition (Mass %)			Size Distribution (µm)	Apparent Density (g/cm^3^)	Flow Ability (s/50 g)
	WC	Co	Cr			
**WC-4Co-10Cr**	86	4	10	15–45	4.98	15
**WC-7Co-7Cr**	86	7	7	15–45	5.02	14
**WC-10Co-4Cr**	86	10	4	15–45	5.11	14

**Table 2:** High-velocity oxygen fuel (HVOF) spraying parameters.

Parameters	Values
Kerosene (L/h)	23
Oxygen (L/h)	885
Carry gas (L/h)	11
Step (mm)	5
Gun Velocity (mm/s)	600
Spraying distance (mm)	350
Feed rate (g)	70

**Table 3:** Main phases content of the as-sprayed WC-Co-Cr coatings (wt.%).

Phase	WC-4Co-10Cr	WC-7Co-7Cr	WC-10Co-4Cr
WC	51.0%	54.4%	63.5%
W_2_C	49.0%	45.6%	36.5%

**Table 4:** Hardness, porosity, and fracture toughness of the three WC-Co-Cr coatings.

Coatings	Hardness (HV0.3)	Porosity (%)	Fracture Toughness (MPa·m^1/2^)
WC-4Co-10Cr	1177 ± 89.1	1.11 ± 0.35	3.12 ± 0.78
WC-7Co-7Cr	1256 ± 87.7	0.55 ± 0.26	4.06 ± 0.56
WC-10Co-4Cr	1250 ± 114.8	0.54 ± 0.28	4.53 ± 0.52

**Table 5:** Corrosion results of the three WC-Co-Cr coatings.

Coatings	Open Circuit Potential (V)	Corrosion Potential (V)	Corrosion Current Density (×10^−6^ A/cm^2^)
WC-4Co-10Cr	−0.46	−0.57	8.97
WC-7Co-7Cr	−0.40	−0.41	3.19
WC-10Co-4Cr	−0.28	−0.35	2.87
16Mnsteel substrate	−0.68	−0.66	38.12

## Data Availability

The data presented in this study are available on request from the corresponding author.
